# A44 A 1000 PATIENT CANADIAN NETWORK FOR AUTOIMMUNE LIVER DISEASE EVALUATION OF CLINICAL AND DEMOGRAPHIC PATTERNS OF AUTOIMMUNE HEPATITIS

**DOI:** 10.1093/jcag/gwac036.044

**Published:** 2023-03-07

**Authors:** C G Plagiannakos, A J Montano-Loza, E Lytvyak, J Pallotta, A L Mason, K M Qumosani, L Worobetz, J A Flemming, J Hercun, C Vincent, A Cheung, T Chen, D Grbic, M G Swain, A Gulamhusein, B E Hansen, G M Hirschfield

**Affiliations:** 1 Toronto Centre for Liver Disease, Toronto Western and General Hospital, University Health Network; 2 Institute of Health Policy, Management, and Evaluation, University of Toronto, Toronto; 3 Division of Gastroenterology and Hepatology, University of Alberta, Edmonton; 4 Department of Medicine, Western University, London; 5 Department of Medicine, University of Saskatchewan, Saskatoon; 6 Medicine and Public Health Sciences, Queen's University, Kingston; 7 Département De Médecins, Centre Hospitalier De l’Université De Montréal, Montréal; 8 Department of Medicine, University of Ottawa, Ottawa; 9 Department of Medicine, McGill University Health Centre, Montréal; 10 Université De Sherbrooke, Sherbrooke; 11 Division of Gastroenterology and Hepatology, University of Calgary, Calgary, Canada; 12 Gastroenterology and Hepatology, Erasmus University Medical Center, Rotterdam, Netherlands

## Abstract

**Background:**

We sought to understand how the demographics of autoimmune hepatitis (AIH) have changed over time in Canada.

**Purpose:**

Using a large multi-centre Canadian cohort of patients with AIH, we describe the trends in patient and disease characteristics at presentation across 30 years of clinical practice.

**Method:**

Patients from the Canadian Network for Autoimmune Liver Disease with a confirmed diagnosis of AIH (simplified score ≥6) were included for analysis. Patients were grouped into five cohorts according to the year of diagnosis (i.e., <2000, 2000-2004, 2005-2009, 2010-2014, ≥2015). Patient demographics and baseline clinical and biochemistry features of disease activity were investigated using Chi-square tests and Kruskal-Wallis tests adjusted for multiple comparisons. Logistic and linear regression models with estimated means were utilized to further investigate relationships with time and to adjust for confounding.

**Result(s):**

1016 patients followed across 10 Canadian health centres with AIH were diagnosed between November 1965 and December 2021. Overall, 76.4% (n=776) of patients were female, and the median age at diagnosis was 46 years (IQR 28.2 - 58.3). Cirrhosis at presentation was seen in 20.6% of patients (n=209). The median age at diagnosis increased significantly from 31.8 years [IQR 17.9 - 46.8] pre-2000 to 54 years [IQR 9.0 - 95.2] after 2014 (p<0.001; Figure 1a). This effect of time persisted after adjusting for sex and cirrhosis status at diagnosis. Female sex and the presence of cirrhosis at diagnosis were factors independently associated with older age at presentation (p<0.0001).

The proportion of patients that presented with cirrhosis at diagnosis increased significantly over calendar time, from 13.7% (n=23) pre-2000 to 30.8% (n=69) after 2014 (p=0.003; Figure 1b). Male sex was independently associated with an increased odds of having cirrhosis at presentation (OR= 1.46, CI 1.02 - 2.07) and higher baseline ALT levels compared to females (p=0.036). The proportion of patients that identified as non-white ethnicity increased significantly from 15.2% (n= 24) pre-2000, to 32% (n= 86) after 2014 (p<0.001, Figure 1b). This effect of time on ethnicity was most pronounced after the year 2010 (OR= 2.32, CI 1.39 - 3.98) and persisted after adjusting for sex. There was no significant pattern of change in sex over calendar time.

**Image:**

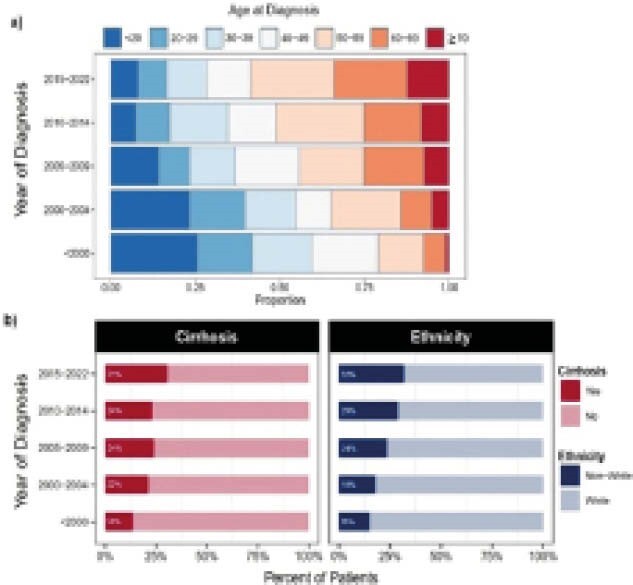

**Conclusion(s):**

In Canada, patients with AIH at presentation are now older, have more advanced disease, and are more ethnically diverse than when compared to 30 years ago.

**Please acknowledge all funding agencies by checking the applicable boxes below:**

Other

**Please indicate your source of funding;:**

industry

**Disclosure of Interest:**

None Declared

